# Active surveillance in intermediate-risk prostate cancer with PSA 10–20 ng/mL: pathological outcome analysis of a population-level database

**DOI:** 10.1038/s41391-021-00448-8

**Published:** 2021-09-10

**Authors:** Peter E. Lonergan, Chang Wook Jeong, Samuel L. Washington, Annika Herlemann, Scarlett L. Gomez, Peter R. Carroll, Matthew R. Cooperberg

**Affiliations:** 1 Department of Urology, Helen Diller Family Comprehensive Cancer Center, University of California, San Francisco, CA USA; 2 Department of Urology, Seoul National University Hospital, Seoul, Republic of Korea; 3 Department of Epidemiology & Biostatistics, University of California, San Francisco, CA USA; 4 Department of Urology, Ludwig-Maximilians-University of Munich, Munich, Germany

**Keywords:** Prostate cancer, Outcomes research

## Abstract

**Background:**

Active surveillance (AS) is generally recognized as the preferred option for men with low-risk prostate cancer. Current guidelines use prostate-specific antigen (PSA) of 10–20 ng/mL or low-volume biopsy Gleason grade group (GG) 2 as features that, in part, define the favorable intermediate-risk disease and suggest that AS may be considered for some men in this risk category.

**Methods:**

We identified 26,548 men initially managed with AS aged <80 years, with clinically localized prostate cancer (cT1-2cN0M0), PSA ≤ 20 ng/mL, biopsy GG ≤ 2 with percent positive cores ≤33% and who converted to treatment with radical prostatectomy from the surveillance, epidemiology, and end results prostate with the watchful waiting database. Multivariable logistic regression was performed to determine predictors of adverse pathology at RP according to PSA level (<10 vs 10–20 ng/mL) and GG (1 vs 2).

**Results:**

Of 1731 men with GG 1 disease and PSA 10–20 ng/mL, 382 (22.1%) harbored adverse pathology compared to 2340 (28%) of 8,367 men with GG 2 and a PSA < 10 ng/mL who had adverse pathology at RP. On multivariable analysis, the odds of harboring adverse pathology with a PSA 10–20 ng/mL (odds ratio [OR] 1.87, 95% confidence interval [CI] 1.71–2.05, *p* < 0.001) was less than that of GG 2 (OR 2.56, 95%CI 2.40–2.73, *p* < 0.001) after adjustment.

**Conclusions:**

Our results support extending AS criteria more permissively to carefully selected men with PSA 10–20 ng/mL and GG 1 disease.

## Introduction

The current treatment paradigm for men with localized prostate cancer involves risk stratification to distinguish men whose disease can be safely managed with active surveillance (AS) from those more likely to benefit from immediate definitive treatment [1–3]. The use of prostate-specific antigen (PSA) as a threshold to define eligibility for AS varies widely, with PSA > 10 ng/mL used to exclude men in certain large AS series [4]. The absolute value of PSA can be a driver of both patient and physician anxiety to offer AS [5]. Contemporary risk stratification tools demonstrate that a higher PSA incurs an increased risk of high-grade cancer; however, it is unclear how applicable a threshold PSA of 10 ng/mL is in an AS population where the volume of biopsy-detected disease is by definition very low.

The National Comprehensive Cancer Network (NCCN) prostate cancer guidelines use Gleason grade group (GG) 1 or 2, <50% biopsy cores positive and PSA 10–20 ng/mL as features that define “favorable intermediate-risk” disease and suggest that AS may be an option for some men within this risk category [6], prompting a pressing need to define AS eligibility more precisely within this group.

Prior analyses using surveillance, epidemiology, and end results (SEER) registry data or similar US datasets to evaluate prostate cancer management and outcomes in AS, have lacked a validated indicator of AS use, and analyses have defined “conservative management” based on the absence of identifiable active treatment, or similar proxies which are not generally adequate. To address this, we utilized the newly released SEER prostate with watchful waiting (WW) database which includes an explicit indicator for AS [7].

In this study, we aimed to determine the risk of pathological upgrading or upstaging according to PSA level (<10 vs 10–20 ng/mL) and grade group (GG) (1 vs 2) in men with intermediate-risk localized prostate cancer who underwent radical prostatectomy (RP) using the SEER-WW database.

## Subjects and methods

Men were identified from the SEER-WW database which includes a dichotomous variable for AS/WW (yes or no/unknown) for men diagnosed with prostate cancer from 18 SEER registries between January 2010 and December 2015 [8]. Over this period, SEER covered approximately 30% of the US population. SEER-WW includes data on the documented initial management intent of the treating physician recorded in the medical record and whether the patient was converted to definitive treatment within one year of diagnosis, which is unique and the primary focus of this dataset compared to prior SEER datasets. The cohort was then restricted to men aged <80 years with clinically localized prostate cancer (cT1-2cN0M0), PSA ≤ 20 ng/mL, biopsy GG ≤ 2 with percent positive cores (PPC) ≤ 33% who underwent RP (*n* = 29,120). Patients with no surgical pathology information were excluded (*n* = 2572), yielding a final cohort of 26,548 for the analyses. The primary outcome was adverse pathology, defined as pathological upgrading (GG ≥ 3) or any upstaging to non-localized disease (≥pT3a).

### Statistical analysis

An inherent limitation of the SEER-WW database is the extent of missing data with only 46% of cases with complete data on basic clinical characteristics for risk stratification [7]. Before granting access to the database, the SEER Program requires investigators to acknowledge that the amount of missing data is considerable. To address this limitation, we performed multiple imputations using the Amelia II, R package (version 1.7.5), which uses an expectation-maximization with bootstrapping algorithm. We have described this in detail in prior publications using the SEER-WW dataset [7, 9]. Briefly, imputed variables were clinical T stage, biopsy GG, PSA, number of positive cores, and PPC. Year of diagnosis, race/ethnicity, age, insurance, marital status, and initial treatment were used as additional covariates for the multiple imputation model. We handled the SEER registry as a cross-sectional variable and year of diagnosis as a time-series variable. PSA was log-transformed. Race/ethnicity, initial treatment, health insurance, and marital status were inputted as nominal variables, and clinical T stage, biopsy GG, and a number of positive cores were inputted as ordered variables in the model. We generated five imputations under 1000 maximum resampling. Owing to a lack of granularity in staging information of T1NOS and T2NOS codes, the data were separated into two datasets (T1 and T2) for the multiple imputation models. These two datasets were then recombined to make the final multiple imputation datasets.

Descriptive statistics were generated to report the demographic, clinical, and pathologic characteristics of the study cohort. Means and standard deviations (SD) were reported for continuous variables. Multivariable logistic regression was performed to determine predictors of adverse pathology at RP. Odds ratios (OR) and 95% confidence intervals (CI) were reported for the regression models. Statistical analyses were performed using R version 3.6 and all p values were 2-sided and *p* < 0.05 was considered statistically significant. This study was granted an exemption and consent was waived by the University of California, San Francisco Institutional Review Board, given the use of publicly available data. This study followed the strengthening the reporting of observational studies in epidemiology (STROBE) reporting guidelines for cohort studies [10].

## Results

In total 26,548 men were included in the final cohort (<80 years, with clinically localized prostate cancer (cT1-2cN0M0), PSA ≤ 20 ng/mL, biopsy GG ≤ 2 with percent positive cores (PPC) ≤ 33% who underwent RP and had surgical pathology available).

Table 1 summarizes baseline characteristics and pathological outcomes of the study cohort according to biopsy GG and PSA level. Of the men with GG 1 disease, 15,301 had a PSA < 10 ng/mL, and 1731 had a PSA 10–20 ng/mL. In men with GG 2 disease, 8367 had a PSA < 10 ng/mL, and 1149 had a PSA 10–20 ng/mL. In the 1731 men who had GG 1 disease and a PSA 10–20 ng/mL, mean (±SD) PSA was 13.1 ng/mL ±2.7, 259 (15.0%) were Black, 1298 (75.0%) had clinical T1 disease and mean was PPC 16.6 ± 8.1. In men diagnosed with GG 2 disease, 8,367 had a PSA < 10 ng/mL, and 1149 had a PSA 10–20 ng/mL. Of the 8367 men with GG 2 disease and a PSA < 10 ng/mL, the mean PSA was 5.4 ng/mL ±1.9, 1038 (12.4%) were Black, 5872 (70.2%) had clinical T1 disease and mean PPC 19.2 ± 7.7.

**Table 1:** Baseline characteristics and pathological results according to biopsy grade group and PSA level.

	Grade group 1			Grade group 2			p*
	PSA < 10 ng/mL (*N* = 15,301)	PSA 10–20 ng/mL (*N* = 1731)	*p*	PSA < 10 ng/mL (*N* = 8367)	PSA 10–20 ng/mL (*N* = 1149)	*p*	
Age at diagnosis, year	59.7 ± 7.0	61.8 ± 6.8	<0.001	61.5 ± 6.9	62.9 ± 6.8	<0.001	<0.001
Race/ethnicity			<0.001			0.004	0.067
White	12,649 (82.7%)	1330 (76.8%)		6813 (81.4%)	889 (77.4%)		
Black	1769 (11.5%)	259 (15.0%)		1038 (12.4%)	176 (15.3%)		
Others/unknown	883 (5.8%)	142 (8.2%)		516 (6.2%)	84 (7.3%)		
Clinical T stage			0.247			0.001	<0.001
T1	11,271 (73.7%)	1298 (75.0%)		5872 (70.2%)	860 (74.8%)		
T2	4030 (26.3%)	433 (25.0%)		2495 (29.8%)	289 (25.2%)		
PSA, ng/mL	5.1 ± 1.9	13.1 ± 2.7	<0.001	5.4 ± 1.9	12.9 ± 2.6	<0.001	<0.001
% Positive cores	17.1 ± 8.0	16.6 ± 8.1	0.026	19.2 ± 7.7	18.9 ± 7.9	0.226	<0.001
Insurance			<0.001			<0.001	0.702
Insured	14,723 (96.2%)	1619 (93.5%)		8072 (96.5%)	1060 (92.3%)		
Medicaid	422 (2.8%)	85 (4.9%)		204 (2.4%)	69 (6.0%)		
Uninsured	156 (1.0%)	27 (1.6%)		91 (1.1%)	20 (1.7%)		
Marital status			<0.001			<0.001	<0.001
Married	12,729 (83.2%)	1341 (77.5%)		6783 (81.1%)	874 (76.1%)		
Single	2572 (16.8%)	390 (22.5%)		1584 (18.9%)	275 (23.9%)		
Pathologic upgrading at RP			<0.001			<0.001	<0.001
No	14,410 (94.2%)	1530 (88.4%)		7002 (83.7%)	875 (76.2%)		
Yes	891 (5.8%)	201 (11.6%)		1365 (16.3%)	274 (23.8%)		
Pathologic upstaging at RP			<0.001			<0.001	<0.001
No	14,149 (92.5%)	1478 (85.4%)		6969 (83.3%)	828 (72.1%)		
Yes	1152 (7.5%)	253 (14.6%)		1398 (16.7%)	321 (27.9%)		
Adverse pathology			<0.001			<0.001	<0.001
No	13,470 (88.0%)	1349 (77.9%)		6027 (72.0%)	672 (58.5%)		
Yes	1831 (12.0%)	382 (22.1%)		2340 (28.0%)	477 (41.5%)		

*PSA* prostate-specific antigen, *RP* radical prostatectomy.

*p value between grade groups.

Compared to men with PSA < 10 ng/mL and GG 2 disease, a significantly smaller proportion of men with PSA 10–20 ng/mL and GG 1 disease experienced pathological upgrading (16.3% vs 11.6%), pathological upstaging (16.7% vs 14.6%) and adverse pathology (28.0% vs 22.1%, all *p* < 0.001). On multivariable analysis, PSA 10–20 ng/mL (OR 1.87, 95% CI 1.71–2.05, *p* < 0.001) had lower odds of adverse pathology at RP than those with GG 2 (OR 2.56, 95% CI 2.40–2.73, *p* < 0.001) after adjustment for age, year of diagnosis, race, clinical stage and PPC (Table 2). As a sensitivity analysis, the interaction between PSA 10–20 ng/mL and GG 2 on the primary endpoint of adverse pathology at RP was found to be not significant (OR 0.90, 95% CI 0.75–1.07, *p* = 0.23) (Supplementary Table 1).

**Table 2:** Multivariable logistic regression of PSA level and biopsy grade group predicting adverse pathology at radical prostatectomy.

	Multivariable odds ratio	95% Confidence interval	*p*
Age, year			
50–59 vs. <50	1.35	1.15–1.60	<0.001
60–69 vs. <50	1.74	1.49–2.06	<0.001
70–79 vs. <50	2.35	1.96–2.82	<0.001
Race/ethnicity			
Black vs. White	1.00	0.91–1.10	0.98
Others/unknown vs. White	1.24	1.09–1.40	0.001
Clinical T stage (T2 vs. T1)	1.03	0.96–1.11	0.34
Year of diagnosis			
2011 vs. 2010	0.92	0.84–1.02	0.104
2012 vs. 2010	0.99	0.90–1.10	0.898
2013 vs. 2010	0.99	0.88–1.10	0.791
2014 vs. 2010	1.10	0.98–1.23	0.099
2015 vs. 2010	1.17	1.05–1.31	0.005
% Positive cores	1.02	1.01–1.02	<0.001
PSA, ng/mL (10–20 vs. <10)	1.87	1.71–2.05	<0.001
Biopsy Grade Group (2 vs. 1)	2.56	2.40–2.73	<0.001

*PSA* prostate-specific antigen.

## Discussion

In this study, we used data from the SEER-WW database, a US population-level database with an explicit indicator of AS/WW, and found that men with GG 1 disease and a PSA of 10–20 ng/mL on AS have a lower risk of adverse pathology at RP compared to men with GG 2 and a PSA < 10 ng/mL. Despite concerns about extending AS criteria to intermediate-risk patients [11], the evidence to support the use of PSA as a threshold to identify appropriate patients and exclude those at higher risk for progression remains unclear. Nonetheless, we found that a larger proportion of men with GG 1 and PSA 10–20 ng/mL received AS than men with GG 2 and PSA < 10 ng/mL in the SEER-WW database.

A cross-sectional study in the National Prostate Cancer Register of Sweden found the use of AS increased from 31% in 2009 to 53% in 2014 in men with GG 1 prostate cancer and PSA 10–20 ng/mL [12]. A further study using this nationwide, population-based cohort compared 5087 men with GG 1 disease and a PSA < 10 ng/mL with men diagnosed with GG 2 disease or PSA 10–20 ng/mL and who underwent RP within 6 months of diagnosis. The outcomes were upgrading from GG 3 to 5 and a compositive outcome of adverse pathology (defined as GG 3–5, extracapsular extension, seminal vesical invasion, or positive lymph nodes). Overall, men with GG 1 disease and a PSA 10–15 ng/mL and PSA density <0.15 ng/mL/cm^3^ did not significantly differ in upgrading or adverse pathology findings compared to men with NCCN low-risk prostate cancer [13]. However, the sample size was small in that analysis compared to our study sample.

Results from 698 patients in Sunnybrook AS cohort 82 patients had a baseline PSA > 10 ng/mL and 157 with a PSA rise to >10 ng/mL during surveillance found that PSA on multivariate analysis was not clearly predictive of future adverse histology at RP [14]. There was also a trend for patients in this cohort with a PSA rising over 10 ng/mL on AS to have a higher Gleason score on follow-up biopsy. Furthermore, a higher incidence of high-grade disease and positive margins at RP among those whose PSA rose over 10 ng/mL on surveillance confirms that PSA monitoring can still yield important information in identifying significant cancers. However, the authors found that no patients who started AS with a PSA over 10 ng/mL had high-grade (Gleason ≥8) disease [14]. This is consistent with prior reports [15, 16] and suggests that for low-risk disease baseline PSA may carry limited prognostic value, but is insufficient to discriminate whether surveillance is a safe strategy.

In addition to upgrading and adverse pathology at RP, longer-term outcomes for men on AS are important, particularly if extending criteria to intermediate-risk patients. Data from the Sunnybrook AS cohort found that 15-year metastasis-free survival was 94% in men with GG 1 regardless of whether PSA was <10 or 10–20 ng/ml vs 84% 15-year metastasis-free survival in those with GG 2 and PSA < 20 ng/ml [17]. These findings also lend further support to the concept of risk stratification in AS based on multivariable instruments rather than binary cut points.

Several limitations are inherent to our analysis, which need to be acknowledged. The SEER-WW dataset does not contain data on PSA density which may reduce the bias of PSA values induced by prostate volume. Recent data suggests that men on AS with GG 2 disease and higher PSA density at baseline have a shorter time to definitive treatment compared to men with GG 1 disease and lower PSA density [18]. AS should be informed by incorporating clinical parameters such as PSA density, PSA kinetics, MRI, and genomic biomarkers into patient-centered decision-making [19]. While the SEER-WW dataset is population-based within SEER registry regions, it does not represent the entire United States and does not include the newly added Massachusetts or Wisconsin SEER registry regions. SEER-WW includes the initial management intent and whether the patient was converted to definitive treatment within 1 year of diagnosis, therefore our results may not be generalizable to a broader population of men on AS where decisions on follow up or treatment should be dynamically informed by the unique longitudinal trajectory of each patient with PSA density, PSA kinetics, MRI and biopsy [18, 19]. Although SEER-WW specifies the initial documented management intent of AS/WW by the treating physician, it does indicate the rationale for subsequent definitive treatment. Our primary outcome was adverse pathology at surgery and is therefore only an intermediate endpoint. The long-term oncologic outcomes of these men are currently unknown. Furthermore, due to imperfect data collection/reporting of pN + in SEER-WW, we were unable to assess pathologic nodal disease at RP as an outcome. Despite this, our study has numerous strengths including a large, racially diverse cohort derived from a dataset with an explicit variable for AS/WW and represents comprehensive nationwide data on current practice patterns on AS.

## Conclusions

In conclusion, our results support extending the criteria for AS to carefully selected men with PSA 10–20 ng/mL for GG 1 prostate cancer and should be accompanied by informed decision-making.

## Supplementary information

Supplementary Data

## Data Availability

Data used in this study are publicly available.
