# Supplementary material for: Active surveillance in intermediate-risk prostate cancer with PSA 10–20 ng/mL: pathological outcome analysis of a population-level database
Source: Prostate Cancer Prostatic Dis. 2021 Sep 10;25(4):690–3. doi: 10.1038/s41391-021-00448-8 (PMC9705238; doi:10.1038/s41391-021-00448-8)
Supplement: Supplementary file 1 — Supplementary Data [file 41391_2021_448_MOESM1_ESM.docx]

**Supplementary Table 1.** Multivariable logistic regression of PSA level and biopsy grade group (GG) predicting adverse pathology at radical prostatectomy including interaction terms for PSA 10-20 ng/mL and biopsy GG 2

|  | Multivariable Odds Ratio | 95% Confidence Interval | *p* |
| --- | --- | --- | --- |
| Age, year |  |  |  |
| 50-59 vs. <50 | 1.35 | 1.15-1.60 | <0.001 |
| 60-69 vs. <50 | 1.74 | 1.48-2.05 | <0.001 |
| 70-79 vs. <50 | 2.35 | 1.96-2.82 | <0.001 |
| Race/Ethnicity |  |  |  |
| Black vs. White | 1.00 | 0.91-1.10 | 0.98 |
| Others/Unknown vs. White | 1.24 | 1.09-1.40 | <0.001 |
| Clinical T Stage (T2 vs. T1) | 1.03 | 0.96-1.11 | 0.35 |
| Year of diagnosis |  |  |  |
| 2011 vs. 2010 | 0.92 | 0.84-1.02 | 0.10 |
| 2012 vs. 2010 | 0.99 | 0.89-1.10 | 0.89 |
| 2013 vs. 2010 | 0.98 | 0.88-1.10 | 0.78 |
| 2014 vs. 2010 | 1.10 | 0.98-1.22 | 0.10 |
| 2015 vs. 2010 | 1.17 | 1.05-1.31 | <0.01 |
| % positive cores | 1.02 | 1.01-1.02 | <0.001 |
| PSA, ng/mL (10-20 vs. <10) | 1.98 | 1.74-2.24 | <0.001 |
| Biopsy Grade Group (2 vs. 1) | 2.60 | 2.43-2.79 | <0.001 |
| Interaction PSA 10-20 x Biopsy Grade Group 2 | 0.90 | 0.75-1.07 | 0.23 |

Abbreviations: PSA, prostate-specific antigen
